# Polymer Labelling with a Conjugated Polymer-Based Luminescence Probe for Recycling in the Circular Economy

**DOI:** 10.3390/polym12061226

**Published:** 2020-05-28

**Authors:** Ivo Kuřitka, Vladimír Sedlařík, Diana Harea, Evghenii Harea, Pavel Urbánek, Ivana Šloufová, Radek Coufal, Jiří Zedník

**Affiliations:** 1 Centre of Polymer Systems, University Institute, Tomas Bata University in Zlin, Trida Tomase Bati 5678, 760 01 Zlin, Czech Republic; sedlarik@utb.cz (V.S.); dyanaharea@gmail.com (D.H.); harea@utb.cz (E.H.); urbanek@utb.cz (P.U.); 2 Department of Physical and Macromolecular Chemistry, Faculty of Science, Charles University in Prague, Hlavova 2030/8, 128 40 Prague, Czech Republic; ivana.sloufova@natur.cuni.cz (I.Š.); radek.coufal@natur.cuni.cz (R.C.); jiri.zednik@natur.cuni.cz (J.Z.)

**Keywords:** polyacetylene, thermoplastics, fluorescence, label, recycling, labelling

## Abstract

In this paper, we present the use of a disubstituted polyacetylene with high thermal stability and quantum yield as a fluorescence label for the identification, tracing, recycling, and eventually anti-counterfeiting applications of thermoplastics. A new method was developed for the dispersion of poly[1-phenyl-2-[p-(trimethylsilyl)phenyl]acetylene] (PTMSDPA) into polymer blends. For such purposes, four representative commodity plastics were selected, i.e., polypropylene, low-density polyethylene, poly(methyl methacrylate), and polylactide. Polymer recycling was mimicked by two reprocessing cycles of the material, which imparted intensive luminescence to the labelled polymer blends when excited by proper illumination. The concentration of the labelling polymer in the matrices was approximately a few tens ppm by weight. Luminescence was visible to the naked eye and survived the simulated recycling successfully. In addition, luminescence emission maxima were correlated with polymer polarity and glass transition temperature, showing a marked blueshift in luminescence emission maxima with the increase in processing temperature and time. This blueshift results from the dispersion of the labelling polymer into the labelled polymer matrix. During processing, the polyacetylene chains disentangled, thereby suppressing their intermolecular interactions. Moreover, shear forces imposed during viscous polymer melt mixing enforced conformational changes, which shortened the average conjugation length of PTMSDPA chain segments. Combined, these two mechanisms shift the luminescence of the probe from a solid- to a more solution-like state. Thus, PTMSDPA can be used as a luminescent probe for dispersion quality, polymer blend homogeneity, and processing history, in addition to the identification, tracing, and recycling of thermoplastics.

## 1. Introduction

Rapid advances in a wide range of polymers have triggered both new challenges and opportunities for recycling plastics. Recycling is essential for the circular economy by allowing the use of recycled raw materials while reducing the need for limited, non-renewable, primary raw materials. High-quality products nevertheless require maintaining the price of the materials without downgrading their high-value properties during the recycling process [1]. However, modern production commonly combines several types of polymers into a single industrial product. Therefore, the identification and traceability of the polymers used in production are key steps.

Various methods for polymer identification by direct analysis of their structure are currently available [2,3], e.g., Mid-infrared Spectroscopy (MIR) [4], Near Infrared Spectroscopy (NIR) [5], and laser-induced breakdown spectroscopy [6], among others. These methods are often combined with automated instrumentations, machine learning procedures, and artificial intelligence applications [7]. In contrast, polymer labelling can be used to track polymers during their life cycle. For such purposes, various probes can be added to polymer compounds, thus labelling polymer brands or even single batches. Polymers can be chemically modified by introducing heavy atoms followed by X-ray fluorescence (XRF) detection, but this process is seldom reported [8] and rather trivial. In contrast, polymer modification using additives is the most commonly applied industrial process of fabrication of plastic materials. Moreover, in this physical modification process, a probe can be easily added to a polymer blend during compounding with other additives.

In this context, the use of probes with various XRF compositions containing elements with an identifiable XRF signature is a highly promising approach for introducing an XRF code-word [9]. A highly sophisticated example found in the literature is the use of unique fillers with spectral features specific to narrow-band visible light fluorescence for anti-counterfeiting and recycling [10,11]. In addition, combining various components allows us to write more than one-bit information into the label [12], and other functions can also be added to the material systems, including thermochromism for dual read tagging [13]. In turn, such particulate materials show rather intriguing chemistry, requiring rare elements for their synthesis. Applying UV-Vis fluorescence inorganic tracers for labelling has similar advantages and drawbacks. Moreover, higher quantities of such tracers are needed for dark (brown and black) than for transparent or white plastics [14,15,16]. Furthermore, a relatively old strategy for labelling polymers using a fluorescent dye probe [17,18,19,20] can be revived for such purposes. Thus, the specific strategy should be chosen based on its inherent advantages and disadvantages.

The main advantages of the traceable molecular probes are their high sensitivity, the low additive concentration required and the wide range of luminescent probes available for polymer labelling [18,19,20]. Conversely, the main disadvantage of luminescent dyes is their migration from the labelled polymer matrix due to diffusion [17,18]. Nevertheless, the use of intrinsically luminescent conjugated polymers such as disubstituted acetylenes could provide new insights into this field of research. The high molecular weight of the conjugated polymer-based luminescence probe brings unexpected advantages thanks to potential interactions between the conjugated polymer probe and the polymer matrix because such interactions can affect emission characteristics, thereby enabling polymer tracking and identification. Moreover, such probes cannot be practically released from polymer blends when compounded properly. Accordingly, the polymer-based fluorescent label may also be used as a unique anticounterfeiting marker and may enrich the current portfolio of markers for plastics, albeit adding only one bit of information. On the other hand, only a highly stable polymer can be a suitable candidate for such a purpose since it has to survive the processing conditions of the polymer matrix.

**Figure 1:**
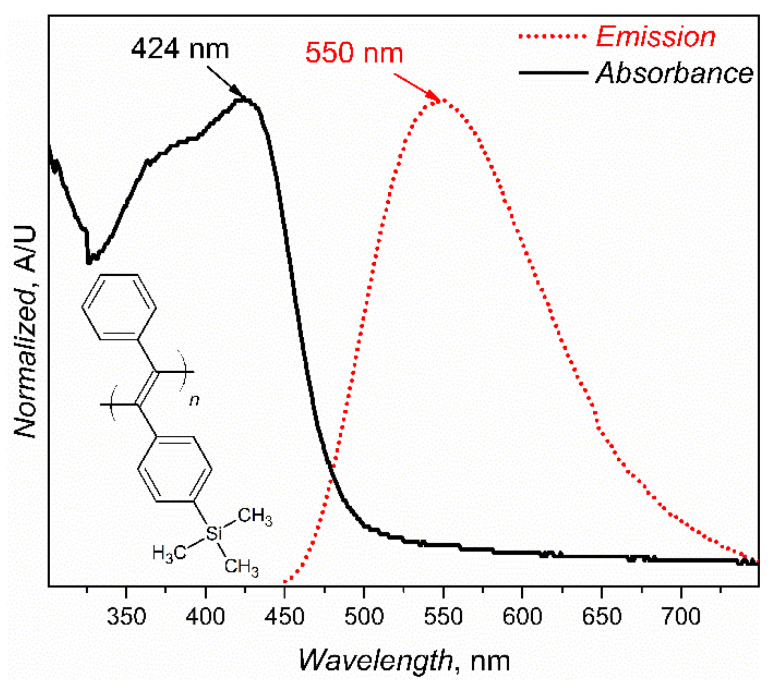
Normalised UV-Vis absorption spectrum and PL emission spectrum of PTMSDPA (pristine additive sample). The PL emission spectrum was recorded at an excitation wavelength *λ_ex_* = 424 nm in a thin film. The formula in the left lower corner of the graph shows PTMSDPA structure.

Based on the above, we report the use of poly[1-phenyl-2-[p-(trimethylsilyl)phenyl]acetylene] (PTMSDPA, the structure is depicted in Figure 1) [21] for labelling polymers for recycling and tracing. PTMSDPA belongs to a conjugated polymer family and is a typical example of disubstituted polyacetylenes [22]. This polymer is widely used for various physical and chemical studies for its high long-term stability [23], gas permeability [24,25], and remarkable photoluminescence [26] in the yellow-green region with a high quantum yield (*Φ* = 0.25 in toluene) [27]. PTMSDPA also has remarkable thermal stability [21]. In this contribution, we report the successful labelling of various representative polymer matrices (polypropylene, PP, low-density polyethylene, LDPE, poly(methyl methacrylate), PMMA, and polylactide, PLA) using a few tens of ppm of this conjugated polymer, showing a correlation between photoluminescence (PL) emission maxima and polymer polarity, glass transition temperature, processing temperature and time, in addition to successfully mimicking polymer recycling by reprocessing. The broad family of thermoplastic polymer resins was limited to the four representative aliphatic polymers to avoid π-π interactions between the polymer probe and matrix in order to keep the interpretational framework tractable in this initial study.

## 2. Materials and Methods

### 2.1. Synthesis and Characterisation of PTMSDPA

PTMSDPA was prepared using a modified procedure [22,25]. Briefly: Syntheses were performed using Schlenk technique under dry and inert conditions (Argon, Linde, Prague, Czech Republic), dissolving 0.5 g TaCl_5_ (ABCR, Schlehert, Germany) in dry, degassed toluene (30 mL), adding 900 µL of tetrabutyltin (Merck KGaA,Sigma-Aldrich, Darmstadt, Germany) in 15 mL of dry, degassed toluene using a stainless-steel capillary, and stirring the mixture at 90 °C (Colour change from yellow to dark brown) for 15 min for ripening. Subsequently, 5 g of 4-(Trimethylsilyl)diphenylacetylene was added to 30 mL of dry, degassed toluene, heating the reaction overnight at 90 °C. The solidified product was dissolved in 1 L of chloroform and precipitated in 2 L of methanol. Dissolution/precipitation was repeated three times to remove the catalyst and tin-based cocatalyst residua. In the last step, the wet product was cut into small pieces using scissors and finally dried in a vacuum oven at 50 °C to constant weight. The yield was virtually quantitative. *M*_w_ 5.2 × 10^6^, *Ð* = 2.8 apparent to polystyrene calibration.

SEC analyses were performed on a Spectra-Physics Analytical HPLC instrument (Thermo Scientific) fitted with two SEC columns Polymer Labs (Bristol, UK) Mixed-A, Mixed-B, and THERMO UV6000 DAD detector, using THF as mobile phase. Absorption spectra were recorded on a Shimadzu UV-2401 PC spectrophotometer using a thin polymer film sample. Luminescence spectra were recorded in a solid-state sample holder on a Fluorolog 3–22 Jobin Yvon Spex instrument (Jobin Yvon Instruments S. A., Palaiseau, France). The corrected emission spectra were recorded using an excitation wavelength *λ*_ex_, equal to the position of the absorption maxima of PTMSDPA (424 nm). Infrared spectra were recorded on a Thermo Scientific Nicolet 7600 FTIR spectrometer equipped with a Spectra Tech InspectIR Plus microscopic accessory using transmission mode on a thin polymer film (128 or more scans at a resolution of 4 cm^−1^). Raman spectra of solid samples were recorded on a DXR Raman microscope (Thermo Scientific) using NIR excitation laser (*λ*_ex_ = 780 nm) and the usual laser power at the sample of 0.1–0.4 mW.

A pristine PTMSDPA film sample for UV-Vis absorbance measurement was prepared on a quartz plate using the drop-cast technique from a chloroform solution (1 wt%).

Photoluminescence of PTMSDPA in solutions was also characterised. The concentration of all solutions was such that the absorbance of the solutions was below 0.05. Cyclohexane of the spectroscopic grade was used. Other solvents were of pro analysis (p.a.) quality. Solvent mixtures of ethyl lactate/chloroform (9:1 vol/vol) and methyl ethyl ketone/chloroform (9:1 vol/vol) were used due to poor solubility of PTMSDPA while cyclohexane and chloroform were used without any additive. Testing of blank samples excluded any solvent impurity luminescence. Excitation wavelength 427 nm was used for the measurement of emission spectra. The excitation spectra were collected at the luminescence emission maximum wavelengths. An FSL 920 fluorimeter (Edinburgh Instruments, Livingston, United Kingdom) was used for these specific measurements.

### 2.2. Preparation of Labelled Polymer Sample Batches and Their Mimicked Recycling

The process of preparation of polymer materials labelled with the PTMSDPA polymer probe merits a detailed description here. An original method for polymer labelling was developed because standard polymer melt mixing failed. This goal was achieved by dipping granules into a diluted PTMSDPA solution in chloroform (2 mg of PTMSDPA in 50 mL). The heterogeneous PTMSDPA solution-soaked or partly dissolved granules were stirred continuously. Then, chloroform was evaporated under airflow. The resulting PMMA and PLA particles were slightly stuck together due to partial dissolution and swelling in chloroform, while PP and LDPE retained the shape of the original particles homogeneously covered by PTMSDPA. The process was completed by drying in vacuum to the constant weight. The parent polymers (PP, LDPE, PLA, and PMMA) differ in density. Therefore, the concentrations of PTMSDPA slightly varied among the samples. The concentration of the PTMSDPA label by weight of material inputs was 32 ppm in PP-, 35 ppm in LDPE-, 23 ppm in PLA-, and 26 ppm in PMMA-labelled polymer sample batches. Furthermore, the dried granules were homogenised using a Brabender Plastograph^®^ mixer at the processing temperature selected for each parent polymer (PP 190 °C, LDPE 140 °C, PLA 230 °C, and PMMA 170 °C) for 5 min. Then, the resulting compounds were crushed into small pieces, thereby preparing specimens for testing. The crushed materials were compression-moulded using stainless steel moulds and poly(ethylene terephthalate) PET or aluminium foil separators at the same respective processing temperatures. The moulding procedure was performed as follows: 5 min preheating followed by 5 min compressing moulding into sheets thick 2 mm or 4 mm, subsequently cooling under 10 MPa pressure for 5 min as the last stage of the process.

All specimens prepared as described above were reprocessed after completing all tests to recycle the material. Specimens prepared in the first processing cycle were ground into small lumps with a size similar to that of the original granules. The resulting materials were again homogenised in the molten state, and specimens were again prepared by compression moulding, using the same equipment and maintaining all processing parameters. Small samples were collected from each cycle for spectroscopic characterisation and archival. The material reprocessing cycle was repeated twice. Thus, these procedures led to two consequent generations of specimens. The total exposure of the material to the processing temperature was 10 min in each processing cycle. All materials from each labelled polymer compound batch were processed and moulded to impart the same processing (temperature) history to the whole sample batch.

Specimens for FT-IR and Raman spectroscopy were prepared as follows: thin foils (hundreds of micrometres) were prepared by the quick and simple compression of a small piece of PTMSDPA-labelled polymer blend at processing temperature.

## 3. Results and Discussion

Simple thermal mixing of PTMSDPA with commonly used polymers, such as polypropylene (PP), low-density polyethylene (LDPE), polymethylmethacrylate (PMMA), and polylactide (PLA), is a major challenge due to the high thermal stability of PTMSDPA. Preparing such blends with the required homogeneity is a key issue, especially when seeking to homogeneously disperse and distribute a small quantity of PTMSDPA in a large amount of a viscous polymer-melt.

The melting temperature of PTMSDPA should be higher than that of most thermoplastic polymers used, according to the literature [21], which was confirmed by DSC and TGA measurements (Supplementary Figures S1 and S2). PTMSDPA showed no significant thermodynamic transition between −50 and 200 °C, thus indicating a glassy state in this temperature range. Weight loss in TGA measurements started above 400 °C (see Supplementary Figure S2). Consequently, blending PTMSDPA with polymer matrices in the processing temperature range of 150–250 °C is a delicate operation. Our attempts to directly mix the polymers in a molten state with PTMSDPA particles either failed in dispersion or led to the macroscopically non-uniform distribution of PTMSDPA additive in the polymer matrix. An original idea, which solved the problem, was to deposit small amounts of PTMSDPA on the surface of the polymer granules (see Section 2.2). The thermal mixing of this material enabled us to label the polymer sample batches.

These labelled polymer compounds were first studied using FT-IR and Raman spectroscopy. Although the foils were still too thick, providing oversaturated spectra in the regions assigned to the polymer matrices, no evidence of the PTMSDPA label was found when using a standard transmission mode due to its low concentration. Selected IR and Raman spectra are shown in Supplementary Figures S3 and S4.

Molecular spectroscopy was also employed to analyse degradation due to processing of polymer matrices. No changes in both FTIR and Raman spectra were identified in PP and LDPE samples at wavenumbers typical for absorption bands of carbonyl groups, which indicate the low degree of degradation of these polymer matrices during processing. While PLA samples were too thick to observe possible polymer degradation in FTIR transmission measurements, the Raman spectra of PLA were recorded. We calculated the integral intensity ratio of the 876 cm^−1^ *ν*(CC) mode of the C-COO group of the polymer chain and the 1457 cm^−1^ band corresponding to the asymmetric bending mode *δ_as_*(CH_3_). This *I*_876_*/I*_1457_ Raman intensity ratio has been reported [28,29] to decrease with the degradation of PLA. In our case, the *I*_876_*/I*_1457_ remained unchanged within the standard deviation after adding PTMSDPA (2.06 ± 0.02 for pristine PLA and 2.05 ± 0.03 for PTMSDPA-PLA). Raman spectra of both PMMA and PTMSDPA-PMMA show characteristic bands [30,31] of amorphous PMMA without any change in mutual band ratios (namely between in-plane bending *δ*(CH_2_) mode at 850 cm^−1^, deformation mode *δ*(CH_3_) at 1453 cm^−1^ and very strong mode at 815 cm^−1^ assigned to in-plane bending *δ*(CO) and *νs*(COC)). Therefore, neither PLA nor PMMA showed any detectable degradation product.

A UV-Vis absorption spectrum and a PL emission spectrum of PTMSDPA are plotted in Figure 1. The pristine PTMSDPA has two clear absorption maxima at the wavelengths *λ_A,max,_*_1_ = 375 nm, represented as a shoulder, and *λ_A,max,_*_1_ = 424 nm. The first absorption maximum can be associated with π-π* transitions on the pendant chromophore side groups, while the second one at the longer wavelengths can be ascribed to π-π* transitions on the frontier π-orbitals delocalised over segments of the main polyacetylene chain. In addition, the only emission maximum of the virgin PTMSDPA is found at the wavelength (*λ_em,pr_*) 550 nm, and only one radiative deexcitation channel is present in the system. These spectral features correspond to the values from the original first report [21], and the *λ_em,pr_* at such a high value is typical of ultrahigh molecular weight PTMSDPA [32].

The polymer prepared in this study is neither stereospecific, in terms of *E/Z* isomerism of substituents in double bonds of the polymer main chain, nor regioregular (head-to-tail). Hence, the polymer coil is not expected to form specific helical structures. The parent polymer matrix affects the luminescence of the PTMSDPA additive. In general, the polarity of chemical species is determined by the relative permittivity (*ε_r_*) of the given material. The relative permittivity of each polymer (at low frequencies, i.e., close to DC extrapolation) is known and tabulated (PP 2.1–2.3, LDPE 2.2–2.4, PLA 3.0–3.2, PMMA 3.2–3.4) [33] or (PP 2.27, LDPE 2.29, PMMA 3.3–3.9) [34] or (PLA 1.7 [35], 2.4 [36], and 2.7 [37]). The relative permittivity of PLA can be lower than that of polyolefines, which is somewhat contra-intuitive considering the presence of polar units in the polymer. Nevertheless, the relative permittivity of PLA strongly depends on the measuring conditions, humidity and water content of the polymer, temperature, isomeric composition of the PLA polymer, crystallinity and tacticity, among other factors [35,37]. Another parameter, which is strongly related to the polymer relaxation, is the glass transition temperature, *T_g_*. Regardless of its variation as a function of the heating/cooling rate, the materials were selected to cover situations above and below *T_g_* when investigated at room temperature. There is a relatively good agreement between all references mentioned above and the typical temperature ranges of *T_g_* of the polymer matrices used in this study (PP −10–0 °C, LDPE −130–(−110) °C or up to −25 °C, PLA 50–70 °C, PMMA 85–160 °C) [33,34,35,36,37]. The complicated case of *T_g_* of polyethylenes is beyond the scope of this article, and readers are referred elsewhere for more details [38]. Briefly, amorphous phases of PP and LDPE matrices are in the rubbery state, while those of PLA and PMMA are in the glassy state at room temperature.

**Figure 2:**
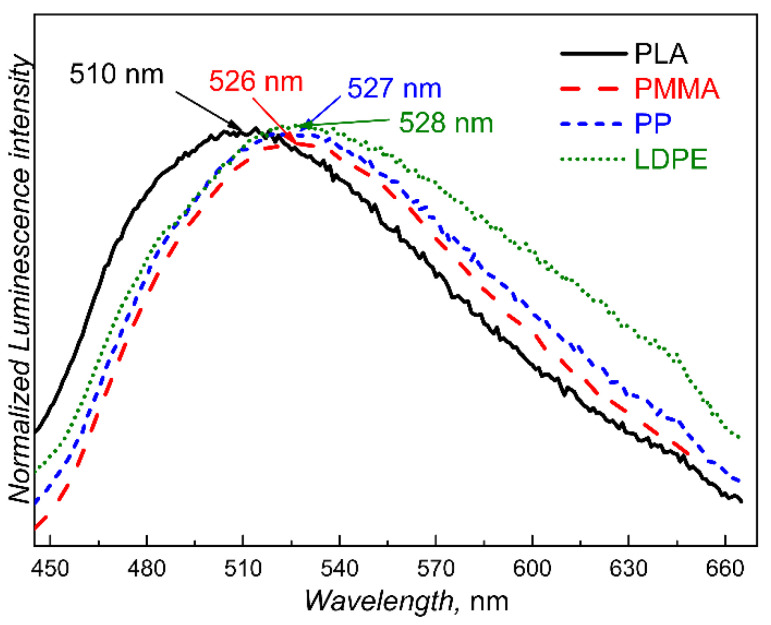
Normalised PL emission spectra of samples of PTMSDPA-labelled polymer compounds prepared in the first processing cycle. Each PL emission spectrum was recorded using an excitation wavelength individually set to the maximum of the excitation spectrum of the respective compound.

Adding such a small amount of PTMSDPA (approximately 20–40 ppm by weight, as indicated in the experimental section) has no practical effect on the parent polymer matrix. Consequently, PTMSDPA does not change either the physical appearance or the physical properties of the respective polymer. The only added feature is photoluminescence under appropriate irradiation. The intense luminescence is visible to the naked eye. We observed a correlation between the position of the PL emission maximum of the PTMSDPA-labelled polymer blend and the tabulated dielectric constants, which was reflected in the redshift with the decrease in polarity, as shown in Figure 2. The PL emission maximum wavelength also correlates with the glassy or rubbery state of the polymer matrix. Both correlations indicate that the ability of the polymer matrix to relax both electronically and vibrationally affects the position of the HOMO and LUMO energy levels in the PTMSDPA and the relaxation of excited states on the backbone chain of the PTMSDPA. Nevertheless, this effect is relatively weak, in comparison to the marked change in the PL emission maximum wavelength of the pristine (solution cast) PTMSDPA material caused by compounding into any of the matrices, which showed relatively large blueshifts in the PL maximum of approximately 22 nm for LDPE and of up to 40 nm for PLA.

The solution cast PTMSDPA films and membranes are known to have excellent gas and vapour permeation thanks to their enormous fractional free volume of approximately 0.26 [24]. The bulky side group substituents impose high steric hindrance on polymer backbone chains, thus inhibiting efficient and dense chain packing. In turn, polymer chains are highly entangled in the solid obtained by freeze-drying from solution, while the chains are isolated and untangled in solution. The randomly entangled chains have either more developed intermolecular π-π interactions or main-chain planarisation, which results in lower electronic transition energies in the solid [26]. In other words, the conjugation length of the segments of the polyacetylene chain is maximally extended, and/or π-stacking occurs when drying the material. Accordingly, the PL emission of the PTMSDPA should be highly sensitive, even to minor changes in the molecular electronic structure. Compounding the PTMSDPA into a viscous polymer melt may enforce conformational changes in polyacetylene chains due to the imposed shear forces, which shorten the average conjugation length of PTMSDPA chain segments. Mixing PTMSDPA into the polymer matrix also disentangles its chains similarly to polymer dissolution. Thus, the effect of intermolecular interactions also weakens, as shown by the blueshift observed between the solution cast thin film and the solution of the PTMSDPA. Fluorescence emission maximum wavelength, *λ*_em,max,film_ = 539 nm shifted by ~31 nm as compared to that of the solution *λ*_em,max,sol_ = 508 nm is reported in the literature [26]. Moreover, slight swelling of the PTMSDPA film by various solvents (alcohols and hydrocarbons) causes a blueshift as well and increases the luminescence intensity drastically as described in the same article. We also observed similar shifts in fluorescence emission maximum for various solvents (Supplementary Figure S5). The solvents were selected due to their similarity with the polymer matrix structural motifs. The normalised solvent polarity (*E_T,n_*) characterising used solvents is derived from solvatochromic effects, the values of *E_T,n_* are taken from reference [39]. While the solid polymer has *λ*_em,max,film_ = 550 nm, the emission maximum wavelength of the solution (*λ*_em,max,sol_) varies with the solvent from *λ*_em,max,sol_ = 505 nm for cyclohexane (*E_T,n_* = 0.006), 509 nm for chloroform (*E_T,n_* = 0.259), 517 nm for methyl ethyl ketone (*E_T,n_* = 0.327)/chloroform mixture up to 520 for ethyl lactate (*E_T,n_* = 0.630)/chloroform mixture. The addition of chloroform improved the solubility of PTMSDPA in methyl ethyl ketone, while it was necessary to dissolve PTMSDPA in ethyl lactate. Unlike in polymer matrix polarity analysis (based on *ε_r_*), the luminescence blueshift in solution correlates perfectly with the decrease in solvent polarity. Thus, a cooperative or competition manifestation of the two mechanisms (i.e., disentanglement and dilution) can explain the blueshift in PL maximum considering the extreme thermal stability of the PTMSDPA. However, quantitative assessment of the contribution of each one of the two mechanisms is impossible at this stage of the research. We were unable to gather enough information on changes in the ratio between 0–0 and 0–1 transitions in the UV-Vis absorption spectrum because UV-Vis absorption measurements are less sensitive than PL measurements by several orders of magnitude, and the concentration of the labelling polymer was only a few tens of ppm. Moreover, the PL emission spectrum was not resolved into more than a single broad emission band either in solid form or in solution. In addition to these experimental obstacles, the analysis of the problem is theoretically complicated due to the undefined *E/Z* isomerism and to the non-regioregularity of the synthesised polymer. Therefore, the advanced interpretational framework, in terms of J- and H- aggregates [40,41], is not used throughout this work.

In short, PTMSDPA can be used as a highly effective probe/sensor for the identification, tracing and anti-counterfeiting of polymer blends. These promising applications require further investigation of our results to confirm the general applicability of the conjugated polymer-based probe reported herein as a probe/sensor with a wide range of industrial applications. Despite the high sensitivity of the luminescent probe and the low amounts of PTMSDPA required, this luminescent probe is relatively expensive, and its synthesis is quite cumbersome, which partly hinders its industrial use. Nevertheless, its effectiveness and efficiency thanks to the long-term stability of the luminescence labelling in plastic waste recycling in the circular economy could be an overwhelming factor, thereby compensating for the high price of this sensor.

**Figure 3:**
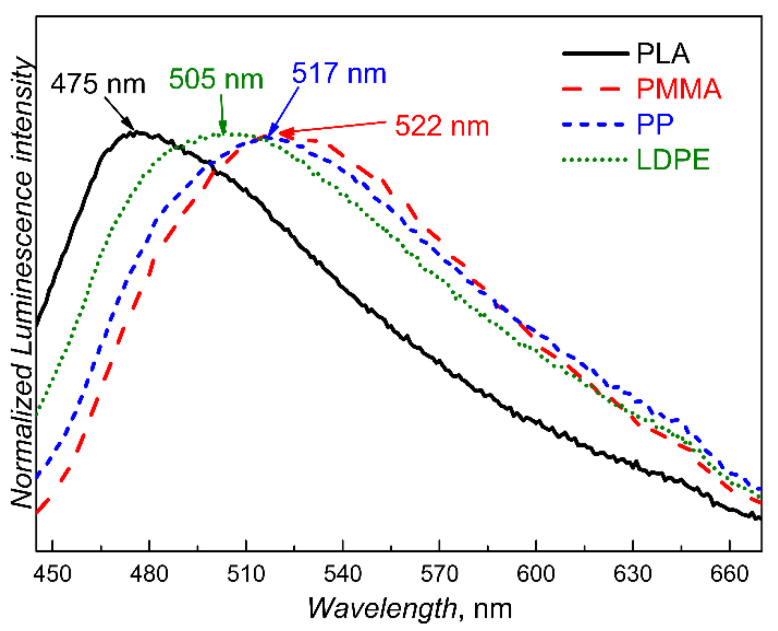
Normalised PL emission spectra of samples of PTMSDPA-labelled polymer compounds in the third processing cycle (i.e., second reprocessing). Each PL emission spectrum was recorded using excitation wavelength set to the maximum of the excitation spectrum of the respective compound individually.

An ideal luminescent-based probe/sensor should survive several processing cycles of the labelled polymer. We performed simulated recycling of PTMSDPA-labelled polymer blends to investigate and demonstrate the potential of our material systems. Reprocessing mimics the effect of recycling in real conditions. Here, we proved the reprocessing effect on the luminescence spectra, as shown in Figure 3. Changes in PL emission maximum wavelength are outlined in Table 1 for all generations of samples. While the effects of polymer matrices on the *ε_r_* and *T_g_* are relatively weak, another correlation was found between the PL emission maximum wavelength, the processing temperature, and cycle number. The higher the cycle number is, the longer the material will be exposed to the processing temperature. The blueshift in the PL emission maximum wavelength from 528 nm to 475 nm is shown in Table 1 following the diagonal path from the upper-left corner to the lower-right corner. This observation supports our interpretation of the blueshift as a result of the shortening of the effective conjugation length of the polyacetylene polymer backbone chain and of the weakening of intermolecular π-π interactions. The higher the temperature and processing time are, the higher the level of the intramolecular disorder will be when introducing conformational defects in the polymer chain, also improving the dispersion of the PTMSDPA in the polymer matrix. Some slight deviations from a strictly monotonous trend may be caused by differences in dispersive effects of the shear forces when mixing various polymer matrices. Nevertheless, these effects on our experimental framework could not be avoided because we were unable to set all parameters freely during sample processing, e.g., the viscosity of the polymer melt depends on the processing temperature. The sample was prepared to optimise mixing by maximising additive dispersion and distribution. It must be noted that the final properties of the recycled materials were left uncontrolled and unoptimised. Thus, the effects of PTMSDPA addition on the properties of a final product shall be thoroughly tested before implementing the marker in any real application.

## 4. Conclusions

Poly[1-phenyl-2-[p-(trimethylsilyl)phenyl]acetylene] (PTMSDPA), a representative member of the disubstituted polyacetylenes family with high thermal stability and quantum yield, was successfully used as a fluorescence label for aliphatic thermoplastics. A new preliminary method was developed for PTMSDPA dispersion in polymer blends of four representative commodity plastics, namely polypropylene, low-density polyethylene, poly(methyl methacrylate), and polylactide. Two reprocessing cycles demonstrated the suitability of the chosen polymer label for identification, tracing, recycling, and eventually anti-counterfeiting applications at very low concentrations of the labelling polymer in matrices. Adding 20–40 ppm (by weight) of PTMSDPA imparts intensive luminescence to labelled polymer blends, and the resulting luminescence is visible to the naked eye. A relatively weak correlation between luminescence emission maxima and polymer polarity and glass transition temperature is explained by the effect of polymer matrix relaxation on the energies of electronic transitions in the PTMSDPA while the polymer polarity is of lesser importance. In contrast, a large blueshift in luminescence emission maxima was shown with the increase in processing temperature and time. Thus, PTMSDPA can also be used as a luminescent probe for dispersion quality, polymer blend homogeneity, and processing history. PTMSDPA chains disentangle during polymer melt homogenisation. Hence, their intermolecular π-π interactions are suppressed, which is a dilution effect. Moreover, the shear imposed on PTMSDPA chains when mixing the viscous polymer matrix melt induces conformational disorders, thereby shortening the average conjugation length of PTMSDPA chain segments. Both mechanisms shift the luminescence of the PTMSDPA probe from a solid- to a more solution-like state.

## Figures and Tables

**Table 1:** Photoluminescence emission maxima observed for all marked polymer samples ordered according to the processing temperature and to the number of the processing cycle.

Polymer Matrix	Processing Temperature (°C)	PL Emission Maximum for Materials Prepared at Processing Cycle, *λ_em,max_* (nm)		
		1st (Primary Processing)	2nd (First Reprocessing)	3rd (Second Reprocessing)
**LDPE**	140	528	512	505
**PMMA**	170	526	525	522
**PP**	190	527	524	517
**PLA**	230	510	480	475

## Supplementary Materials

The following are available online at https://www.mdpi.com/2073-4360/12/6/1226/s1. The file contains four figures. Figure S1: DSC record of PTMSDPA polymer in a nitrogen atmosphere. Figure S2: TGA record of PTMSDPA polymer in a nitrogen atmosphere and air. Figure S3: FT-IR spectra of PTMSDPA labelled PP and LDPE. Figure S4: Raman spectra of PTMSDPA labelled polymers. Figure S5: Normalised PL excitation and emission spectra of PTMSDPA dissolved in various solvents.
