# Supplementary material for: Polymer Labelling with a Conjugated Polymer-Based Luminescence Probe for Recycling in the Circular Economy
Source: Polymers (Basel). 2020 May 28;12(6):1226. doi: 10.3390/polym12061226 (PMC7362226; doi:10.3390/polym12061226)
Supplement: Supplementary file 1 [file polymers-12-01226-s001.pdf]

polymers

MDPI

Electronic Supplementary Information - ESI

Polymer labelling with a conjugated polymer-based luminescence probe for recycling in the circular economy

Ivo Kuřitka [1,*], Vladimír Sedlařík [1], Diana Harea [1], Evghenii Harea [1], Pavel Urbánek [1], Ivana Šloufová [2], Radek Coufal [2] and Jiří Zedník [2]

[1] Centre of Polymer Systems, University Institute, Tomas Bata University in Zlin, trida Tomase Bati 5678, Zlin 760 01, Czech Republic; sedlarik@utb.cz (V.S.); dyanaharea@gmail.com (D.H.); harea@utb.cz (E.H.); urbanek@utb.cz (P.U.)

[2] Department of Physical and Macromolecular Chemistry, Faculty of Science, Charles University in Prague, Hlavova 2030/8, Prague 128 40, Czech Republic; ivana.sloufova@natur.cuni.cz (I.S.); radek.coufal@natur.cuni.cz (R.C.); jiri.zednik@natur.cuni.cz (J.Z.)

* Correspondence: kuritka@utb.cz

PTMSDPA stands for poly[1-phenyl-2-[p-(trimethylsilyl)phenyl]acetylene] anywhere.

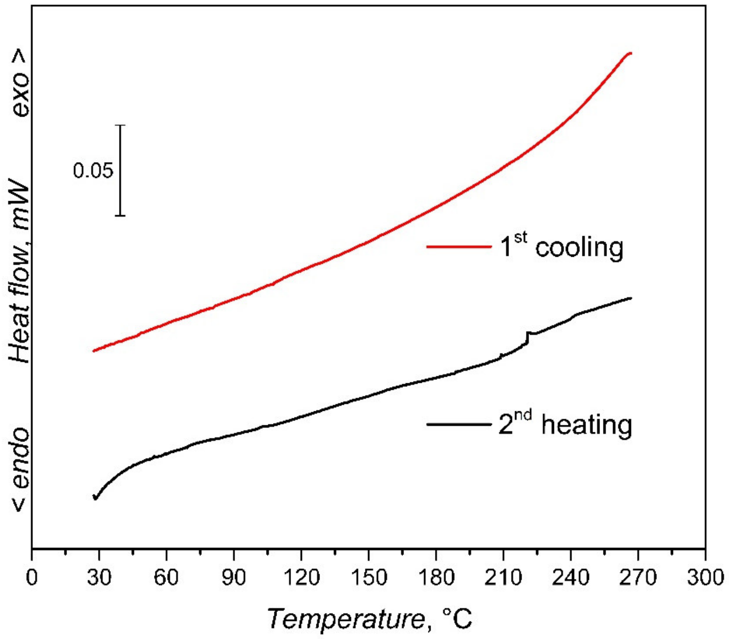

Figure S1 DSC record of **PTMSDPA** polymer in a nitrogen atmosphere

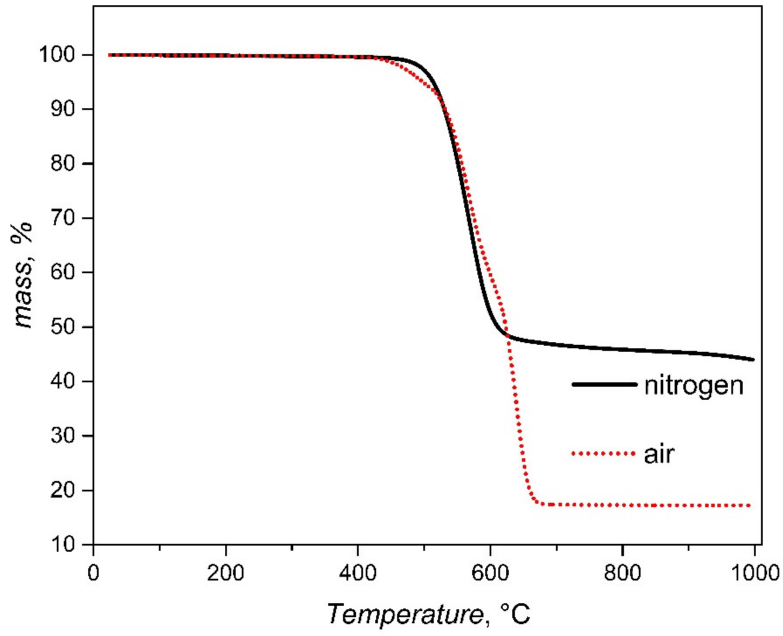

Figure S2 TGA record of **PTMSDPA** polymer in a nitrogen atmosphere and air

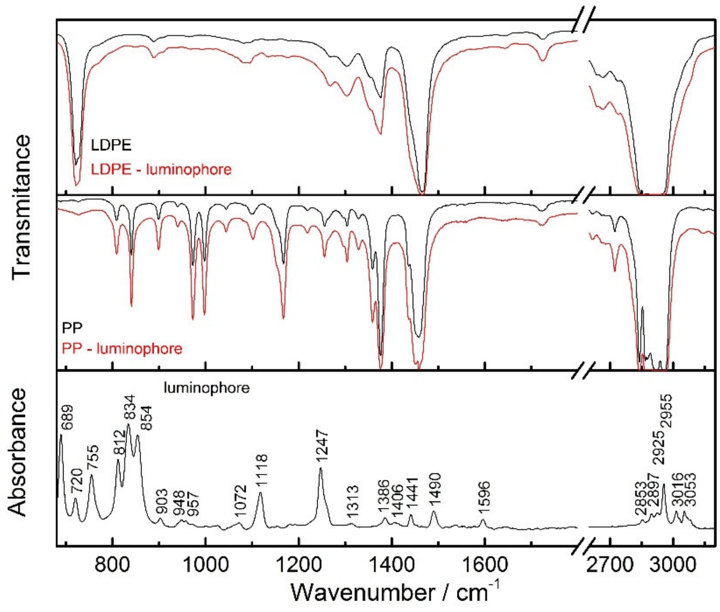

Figure S3 FT-IR spectra of PTMSDPA-labelled PP and LD-PE

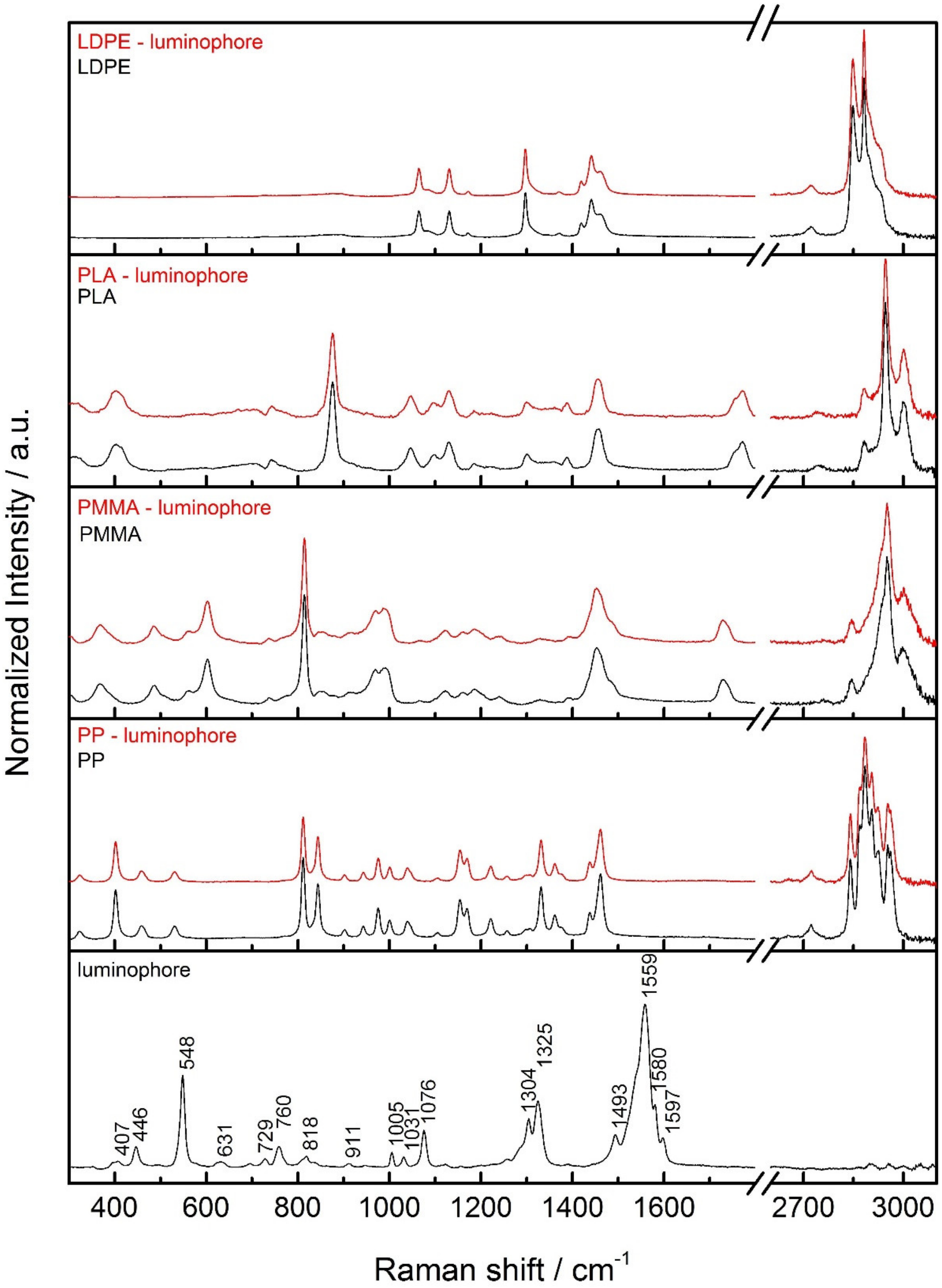

Figure S4 Raman spectra of **PTMSDPA** labelled polymers

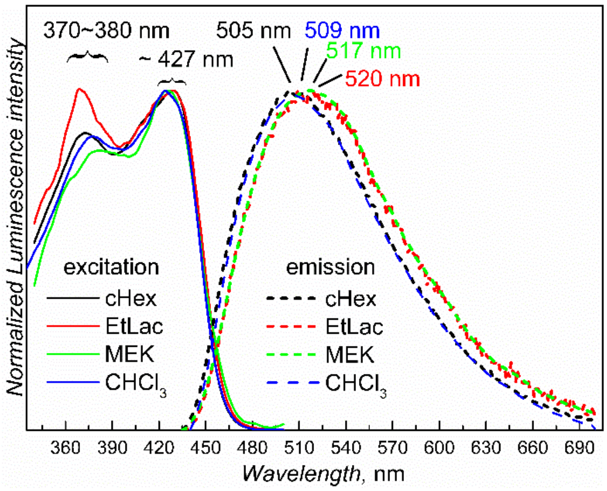

Figure S5 Normalised PL excitation and emission spectra of **PTMSDPA** dissolved in various solvents. The spectra measured for the cyclohexane solution is denoted cHex, the ethyl lactate/chloroform mixture solution is denoted EtLac, the methyl ethyl ketone/chloroform mixture solution is denoted MEK, and the chloroform solution is denoted $CHCl_3$ in the figure.
